# Binding of *Trichinella spiralis* C-type lectin with syndecan-1 on intestinal epithelial cells mediates larval invasion of intestinal epithelium

**DOI:** 10.1186/s13567-023-01217-2

**Published:** 2023-10-02

**Authors:** Zhen Wang, Qi Qi Lu, Min Min Weng, Yang Li Li, Lu Lu Han, Yan Yan Song, Yu Long Shi, Ruo Dan Liu, Jing Cui, Zhong Quan Wang

**Affiliations:** Department of Parasitology, Medical College, Zhengzhou University, Zhengzhou, 450052 China

**Keywords:** *Trichinella spiralis*, C-type lectin, syndecan-1, intestinal epithelium, invasion

## Abstract

C-type lectin (CTL) is a protein that binds to saccharides and plays an important role in parasite adhesion, host cell invasion and immune evasion. Previous studies showed that recombinant *T. spiralis* C-type lectin (rTsCTL) promotes larval invasion of intestinal epithelium cells (IEC), whereas anti-rTsCTL antibodies inhibits larval invasion. Syndecan-1 (SDC-1) is a member of the heparan sulfate proteoglycan family which is mainly expressed on the surface of IEC and in extracellular matrices where they interact with a plethora of ligands. SDC-1 has a principal role in maintaining cell morphogenesis, establishing cell–cell adhesions, and regulating the gut mucosal barrier. The aim of this study was to investigate whether rTsCTL binds to SDC-1 on IEC, and the binding of rTsCTL with SDC-1 promotes larval invasion and its mechanism. IFA results show that rTsCTL and SDC-1 co-localized on Caco-2 cell membrane. GST pull-down and Co-IP verified the direct interaction between rTsCTL and SDC-1 on Caco-2 cells. qPCR and Western blotting revealed that rTsCTL binding to SDC-1 increased the expression of SDC-1 and claudin-2, and reduced the expression of occludin and claudin-1 in Caco-2 cells incubated with rTsCTL via the STAT3 pathway. β-Xyloside (a syndecan-1 synthesis inhibitor) and Stattic (a STAT3 inhibitor) significantly inhibited rTsCTL binding to syndecan-1 in Caco-2 cells and activation of the STAT3 pathway, abrogated the effects of rTsCTL on the expression of gut tight junctions, and impeded larval invasion. The results demonstrate that binding of rTsCTL to SDC-1 on Caco-2 cells activated the STAT3 pathway, decreased gut tight junction expression, damaged the integrity of the gut epithelial barrier, and mediated *T. spiralis* invasion of the gut mucosa. TsCTL might be regarded as a candidate vaccine target against *T. spiralis* invasion and infection.

## Introduction

*Trichinella spiralis*, the main causative agent of trichinellosis, is a foodborne zoonotic nematode that infects more than 150 species of mammals, and humans [1]. *T. spiralis* infection is hazardous to animal and human health in most regions in the world [2]. Human trichinellosis is caused by eating raw or semi-cooked meat contaminated with *T. spiralis* infectious larvae (muscle larvae, ML). In China, eight outbreaks of human trichinellosis with 479 cases and 2 deaths were reported from 2009 to 2020, and seven outbreaks (87.50%) were involved in the ingestion of raw or poorly cooked pork; pork from domestic hogs is the principal source of human *T. spiralis* infection [3]. *T. spiralis* infection in food animals is also a major risk to meat food safety. Hence, preventive vaccines to control and block *T. spiralis* infection in food animals is needed [4, 5].

After encapsulating *T. spiralis* ML in skeletal muscles, they are digested by gastric juice. The ML are released from their collagen capsules and activated into intestine infectious larvae (IIL), and the IIL invade the gut epithelium where they undergo 4 moltings to mature to adult worms (AW) at 31 h post-infection (hpi) [6]. The male and female AW mate immediately in the gut mucosal epithelia, and deposit the newborn larvae (NBL), which penetrate the gut mucosal blood capillary into the bloodstream, invading skeletal muscles where they develop to the encapsulated ML and finish the life cycle of *T. spiralis* [7]. The gut epithelium is the first site of *T. spiralis* invasion and infection of the host, and it is also the first natural physical barrier for the host to resist *T. spralis* infection [8, 9]. Larval recognition and invasion of the host’s intestinal epithelial cells (IEC) is a key step for *T. spiralis* infection [10], but the larval invasive mechanism has not been fully elucidated up to now [11, 12].

C-type lectin (CTL) is a protein superfamily that selectively binds to saccharides in the presence of Ca^2+^ and widely exists in vertebrates and invertebrates [13]. CTL has one or more CLECT domains, generally consisting of 110–130 amino acids. It contains a super-secondary structure folded into 6 or 7 antiparallel β-chains and 2 α-helices, which is a typical double-loop structure [14]. Except for carbohydrates, the CTL has the capacity to recognize various ligands (lipids, proteins, and uric acid crystals). Previous studies showed that helminth-derived lectins play an important role in parasite adhesion, cell invasion and immune escape [15]. *Toxoplasma gondii* lectin-CD209 mediates the host’s cell invasion, and the parasite invasion and burden are obviously inhibited by ligand mimicking-oligosaccharides [16]. *Cryptosporidium* C-type lectin mediates *Cryptosporidium parvum* attachment and infection to IEC by interacting with heparan sulfate proteoglycans (HSPG); the infection is inhibited by glycosaminoglycans [17].

In our previous studies, a novel *T. spiralis* C-type lectin domain-containing protein (TsCTL; GenBank: KRY42391.1) from NBL soluble proteins was identified by immunoproteomics [18]. TsCTL has a C-type lectin carbohydrate recognition domain (CRD) which was expressed in various *T. spiralis* stages (ML, IIL, AW and NBL), and highly expressed at the IIL stage. rTsCTL was specifically bound with IEC and the gut epithelium. rTsCTL obviously facilitated larval invasion of IEC, whereas anti-rTsCTL antibodies and mannose significantly suppressed larval invasion [19, 20]. However, the kind of IEC proteins binding to rTsCTL have not yet been identified, and the molecular mechanism of TsCTL mediating larval invasion of the gut epithelium is not clear.

Heparan sulfate proteoglycan (HSPG) is a kind of sugar complex composed of core protein and one or more heparan sulfate sugar chains covalently linked to the core protein. According to the location of the cell microenvironment, HSPG can be divided into the cell membrane and extracellular matrix proteoglycans. Syndecan-1, 2, 3, 4 and glypican-1, 2, 3, 4, 5, 6 are located in the cell membrane. Perlecan and agrin are located in the matrix [21]. Syndecan-1 (SDC-1) is a type I integral membrane proteoglycan containing both heparan sulfate and chondroitin sulfate, which connects the cytoskeleton and intercellular matrix. SDC-1 is mainly expressed on the epithelial surface and extracellular matrix where they interact with a plethora of ligands [22]. SDC-1 plays a major role in maintaining cell morphology, accelerating tissue repair, establishing cell-cell adhesion and regulating the intestinal mucosal epithelium barrier [23]. During the progression of breast cancer, the in vitro and in vivo knockdown of galectin-3 leads to the reduction of syndecan-1 on the cell surface in 4T1 cells [24]. Syndecan-1 significantly regulates the expression of gut epithelial tight junctions (TJs; ZO-1 and occludin) by activating STAT3, which directly binds to the promoter regions of ZO-1 and occludin. Therefore, we assumed that TsCTL can bind with syndecan-1 to the gut epithelium cells, which in turn activates STAT3, reduces TJ expression, impairs the integrity of the gut epithelium barrier, and thus, promotes *T. spiralis* invasion of the gut epithelium [25].

The aim of this study was to investigate whether TsCTL binds to SDC-1 on IEC and whether the binding of TsCTL to SDC-1 mediates larval invasion and the mechanisms involved.

## Materials and methods

### *Trichinella* species, plasmids, bacterium strain and cells

*Trichinella spiralis* (ISS534) was collected from an infected pig in Henan province, China and preserved by serial passage in BALB/c mice in our department [26]. The expression plasmid pGEX-4T-1 and the *Escherichia coli* Origami (DE3) strain used in this study were stored in our laboratory. Human colon cancer epithelial cell line Caco-2 cells were purchased from the Cell Bank of the Chinese Academy of Sciences. All experimental projects were authorized by the Life Science Ethics Committee of Zhengzhou University (No. ZZUIRB GZR 2022-1317).

### Worm collection and soluble antigen preparation

The ML was collected by artificially digesting *T. spiralis-*infected mouse muscles at 35 days post-infection (dpi). The IIL were obtained from the small intestine of infected mice at 6 hpi [27]. The IIL somatic soluble antigens (SAg) were prepared as described before [28, 29].

### Cloning, expression and identification of TsCTL

Recombinant plasmid pQE-80L/TsCTL constructed in our laboratory was used as an amplification template [20]. The TsCTL gene was amplified by PCR using specific primers with BamHI and EcoRI restriction sites (in bold) as follows: 5′-C**GGATCC** AACCGTTTTCCGTGCCGTATCAAAT-3′ and 5′-GCGC**GAATTC**TCACTCCAACGAA TGACAAATTC-3′. After amplification, the PCR product was cloned into the expression plasmid pGEX-4T-1 and recombinant pGEX-4T-1/TsCTL was transformed into *Escherichia coli* Origami (DE3) for rTsCTL protein expression. The rTsCTL was expressed by inducing with 0.1 mM IPTG at 25 °C for 16 h [30]. rTsCTL was purified using a GST fusion protein purification kit (Sangon Biotech, Shanghai, China) and identified by SDS-PAGE and Western blot [28, 31].

### Preparation of polyclonal antibodies against rTsCTL and GST tag protein

Forty female mice were divided into two groups (20 animals each). Each group of mice was injected subcutaneously with 20 µg rTsCTL or GST tag protein emulsified with complete Freund’s adjuvant, and followed by two boost immunizations with 20 µg rTsCTL or GST tag emulsified with incomplete Freund’s adjuvant at a 2-week-interval [32]. At 2 weeks after the third injection, the tail blood from all immunized mice was recovered to prepare anti-rTsCTL serum or anti-GST serum; pre-immune serum was also obtained to use as a negative serum control [33, 34].

### Western blotting analysis of rTsCTL

The purified rTsCTL were separated on 10% SDS-PAGE [35]. The proteins were transferred onto nitrocellulose (NC) membrane (Millipore, USA) in the wet transfer cell (Bio-Rad, USA) [36]. The membrane was blocked using 5% skimmed milk in Tris-buffered saline containing 0.05% Tween (TBST) at 37 °C for 2 h, and cut into strips. The strips were incubated with diverse serum (1:100; anti-rTsCTL serum, infection serum, anti-GST serum and normal serum) at 37 °C for 2 h. After being washed using TBST, the strips were incubated at 37 °C for 1 h with HRP conjugated-anti-mouse IgG (1:10 000; Southern Biotech, USA). After washes, the color was developed with 3,3′-diaminobenzidine tetrahydrochloride (DAB; Sigma-Aldrich) [37, 38].

### Cell viability assayed by CCK-8 test

The effect of rTsCTL, IIL SAg and GST tag protein on Caco-2 cell viability was ascertained by a CCK-8 assay kit (Solarbio, China). Cells were cultivated in a Dulbecco modified eagle medium (DMEM; Servicebio, Wuhan, China) supplemented with 4 mM glutamine, 1 mM sodium pyruvate, 20 mM Hepes, 0.1 U/mL bovine insulin (Sigma), 100 U/mL penicillin, 100 µg/mL streptomycin, and 10% fetal bovine serum (Gibco). Caco-2 cells were cultured in a 96-well plate at 37 °C, 5% CO_2_ until grown to confluence. Various concentrations (0–30 µg/mL) of rTsCTL, IIL SAg and GST tag protein were added into the medium, and cultured for 24 h, Then, 10 µL of CCK-8 solution was added to each well of the culture plate and incubated for 1 h. Absorbance at 450 nm was measured with a plate reader (Tecan, Switzerland) [39].

### Immunofluorescence assay (IFA)

The co-localization of rTsCTL and syndecan-1 in Caco-2 cells was investigated by IFA as reported before [35, 40]. Caco-2 cells were cultivated in a 6-well culture plate until confluence [6]. Caco-2 cells were incubated with rTsCTL (5 µg/mL) at 37 °C for 2 h. After being washed three times with PBS, the cells were fixed with 4% paraformaldehyde for 20 min and subsequently blocked with 5% goat serum at 37 °C for 2 h. Caco-2 cells were probed by anti-rTsCTL serum (1:10) and anti-syndecan-1 antibody (1:150, Abmart, Shanghai, China). Alexa Fluor 488-conjugated anti-mouse IgG and CY3-conjugated anti-rabbit IgG (1:100; Servicebio) were used as the secondary antibody. Cell nuclei were stained blue with 4′,6-diamidino-2-phenylindole (DAPI; Solarbio, Beijing, China), and the cells were observed by fluorescence microscopy [6, 41].

### GST pull-down test

To investigate the interaction of TsCTL and syndecan-1 in Caco-2 cell protein, the GST pull-down test was performed as described previously [42]. rTsCTL carrying the GST tag protein was first incubated and bound with GST resins (Sangon Biotech, Shanghai, China) for 2 h at 4 ℃, and the GST resins were washed and maintained in binding buffer (4.2 mM Na_2_HPO_4_, 2 mM KH_2_PO_4_, 140 mM NaCl, and 10 mM KCl). For the GST pull-down assay, Caco-2 cells were lysed on ice for 30 min with RIPA buffer (40 mM Tris–HCl pH 7.6, 150 mM NaCl, 2 mM EDTA, 10% glycerol, 1% Triton X-100, 0.2% SDS, 1 mM PMSF and a protease inhibitor cocktail), and cell debris was removed by centrifugation at 12 000 × *g* for 20 min at 4 °C. The lysate of Caco-2 cells was incubated at 4 °C for 2 h with GST-rTsCTL pre-immobilized to GST resins. The unbound proteins were washed away and the binding of rTsCTL to syndecan-1 in Caco-2 cells was identified by Western blot analysis [43, 44]. Additionally, the GST tag protein and blank beads were set as negative controls and 1/10 volume of lysates of Caco-2 cell proteins was used as the input of the proteins. On Western blot analysis, rabbit anti-human syndecan-1 antibody (1:1000; Abmart, Shanghai, China), mouse anti-rTsCTL serum and anti-GST serum (1:100 dilutions) prepared in our laboratory were used as the primary antibodies, HRP-labeled goat anti-rabbit IgG and goat anti-mouse IgG (1:10 000 dilution, Southern Biotech., USA) served as the second antibodies. The membrane was incubated at 37 °C for 1 h and washed with TBST, and then, Omni-ECL reagents (Epizyme, Shanghai, China) were used to visualize the reactive bands [6, 45].

### Co-immunoprecipitation (Co-IP)

Caco-2 cells were incubated with rTsCTL and GST-tag proteins (5 µg/mL) at 37 ℃ and 5% CO_2_ for 2 h. After incubation, Caco-2 cells were lysed on ice for 30 min, and cell debris was removed by centrifugation at 12 000 × *g* for 40 min at 4 °C. The protein A/G plus-agarose immunoprecipitation kit (Santa Cruz Biotechnology, USA) was used for Co-IP according to the manufacturer’s instructions. The protein A/G agaroses were incubated with murine anti-rTsCTL serum, anti-GST serum and normal IgG at 4 ℃ rotation for 1 h, followed by the addition of the Caco-2 cells lysates described above and rotation for 4 h. After washes and denaturation, proteins interacting with rTsCTL were collected for Western blot analysis [46].

### Real-time quantitative PCR (qPCR)

To assess the transcription levels of syndecan-1, tight junction proteins (TJ; occludin, claudin-1 and claudin-2) in Caco-2 cells treated with rTsCTL and inhibitors, qPCR was performed as reported before [47]. Briefly, after incubation with GST-rTsCTL (5 µg/mL) at 37 ℃ for 2 h, total RNA of Caco-2 cells was extracted using Trizol reagent (Invitrogen, USA) and reversely transcribed into cDNA as templates for qPCR. qPCR was performed using the SYBR Green PCR master mix (Servicebio) in the ABI Prism 7500 Fast Sequence Detection System (Applied Biosystems, USA) [48]. Transcription levels of syndecan-1, occludin, claudin-1 and claudin-2 were ascertained by qPCR with specific primers (Table 1). The relative transcription levels were normalized by subtracting transcription of Caco-2 cell housekeeping gene β-Actin (GenBank: BC013380) and then calculated on the basis of the 2^−ΔΔCt^ method [49, 50]. Each experiment had three replicates. Furthermore, Caco-2 cells were also first treated with 5 mM β-xyloside (syndecan-1 synthesis inhibitor; Yuanye, Shanghai, China) and 10 µM Stattic (a potent STAT3 inhibitor; MCE, China) for 4 h at 37 ℃, and then incubated with 5 µg/mL GST-rTsCTL for 2 h [17]. The transcription levels of related genes (syndecan-1, occludin, claudin-1 and claudin-2) in Caco-2 cells treated with inhibitors were also assessed by qPCR.

**Table 1:** **Specific primer sequences of Caco-2 cells for qPCR**

Genes	Primers	Sequences (5′ end to 3′ end)
Syndecan-1	Forward primer	TCCTGGACAGGAAAGAGGTGCT
	Reverse primer	TGTTTCGGCTCCTCCAAGGAGT
Occludin	Forward primer	ATGGCAAAGTGAATGACAAGCGG
	Reverse primer	CTGTAACGAGGCTGCCTGAAGT
Claudin-1	Forward primer	GTCTTTGACTCCTTGCTGAATCTG
	Reverse primer	CACCTCATCGTCTTCCAAGCAC
Claudin-2	Forward primer	GTGACAGCAGTTGGCTTCTCCA
	Reverse primer	GGAGATTGCACTGGATGTCACC
β-Actin	Forward primer	CACCATTGGCAATGAGCGGTTC
	Reverse primer	AGGTCTTTGCGGATGTCCACGT

### Western blotting of syndecan-1, TJ proteins and signaling pathway

Caco-2 cells were treated with 5 mM β-xyloside or 10 µM Stattic for 4 h at 37 ℃, and then were incubated with GST-rTsCTL (5 µg/mL) at 37 ℃ for 2 h [17]. The Caco-2 cells were lysed in RIPA buffer, ultrasonicated in an ice bath for 30 s and centrifuged at 12 000 × *g* for 15 min to remove any cell fragments. The cell soluble proteins were separated by 10% SDS-PAGE and transferred onto a PVDF membrane (Millipore, USA) in the wet transfer cell (Bio-Rad, USA). The membrane was blocked with 5% skim milk in TBST at 37 °C for 2 h and incised into strips. The strips were probed with antibodies against syndecan-1 (1:1000), total STAT3 (t-STAT3; 1:1000), phosphorylated STAT3 (p-STAT3; 1:5000, Abmart, China), occludin (1:500), claudin-1 (1:200), claudin-2 (1:200) (ThermoFisher, USA), and anti-β-Actin antibody (1:1000) overnight at 4 °C [6]. After washes with TBST three times, the strips were incubated at 37 °C for 1 h with HRP-conjugated anti-rabbit IgG (1:10 000; Southern Biotech). And then, Omni-ECL reagents (Epizyme, Shanghai, China) were used to visualize the reactive bands, and the relative intensities of each band were analyzed using the Image J software (National Institutes of Health, USA) [36].

### The in vitro larval invasion test

To investigate whether TsCTL accelerates larval invasion of the gut epithelium, an in vitro invasion test was conducted as previously reported [10, 51]. Briefly, the ML were activated into the IIL with 5% swine bile at 37 °C for 2 h, and different doses of rTsCTL (5, 10, 15, and 20 µg/mL) and one hundred IIL were added to semisolid medium. Simultaneously, the same doses of GST tag protein were used as a negative protein control. After culture at 5% CO_2_ at 37 °C for 2 h, larval intrusion into the Caco-2 cell monolayer was examined by microscopy. The invaded IIL were active and migratory within the cell monolayer, while the non-invaded IIL were coiled on the surface of the monolayer [8]. To evaluate the suppressive role of β-xyloside and Stattic on larval invasion, Caco-2 cells were first incubated with different doses of β-xyloside and Stattic at 37 ℃ for 4 h, and then the IIL were added onto the confluent Caco-2 monolayer in semisolid medium. After culture at 5% CO_2_ at 37 °C for 2 h, larval invaded into Caco-2 cells was examined by microscopy. Moreover, for further analyzing whether β-xyloside and Stattic could inhibit and abrogate the rTsCTL facilitative on larval invasion, Caco-2 cells were pre-incubated with different doses of β-xyloside and Stattic at 37 ℃ for 4 h, and then rTsCTL (10 µg/mL) and IIL were added onto the monolayer. After culture at 5% CO_2_ at 37 °C for 2 h, larval intrusion of Caco-2 cells was observed under microscopy [52].

### Statistical analysis

All the data are analyzed by the software including SPSS 21.0 and GraphPad Prism, and the results are presented as means ± standard deviation (SD). One way ANOVA, *t*-test, chi-square test, and linear regression were used for statistical analysis in this study. *P* < 0.05 denotes that the difference is considered statistically significant.

## Results

### Expression and identification of rTsCTL

**Figure 1:**
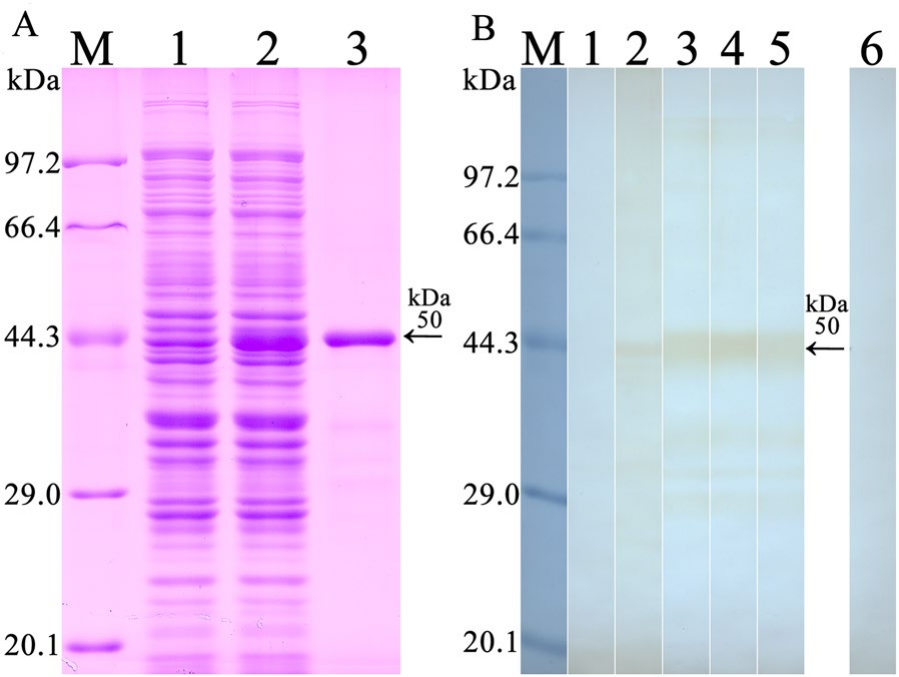
**Identification of rTsCTL. A** SDS-PAGE analysis of rTsCTL. Lane M: protein marker; Lane 1: lysate of bacteria carrying pGEX-4T-1/TsCTL prior to induction; Lane 2: lysate of bacteria carrying pGEX-4T-1/TsCTL after induction; Lane 3: purified rTsCTL (black arrow). **B** Western blotting analysis of rTsCTL. The lysates of bacteria carrying pGEX-4T-1/TsCTL prior to induction (lane 1) was not recognized by infection serum; lysate of induced bacteria carrying pGEX-4T-1/TsCTL (lane 2), purified rTsCTL (lane 3–6) were recognized with infection serum (lane 2, 3), anti-rTsCTL serum (lane 4) and anti-GST-tag serum (lane 5), but not by normal serum (lane 6).

The complete TsCTL coding sequence consisted of 627 bp encoding 208 amino acids (aa), with a molecular weight (MW) of 24 kDa. SDS-PAGE results show that the GST-rTsCTL protein was successfully expressed, the MW of the purified rTsCTL with GST tag was 50 kDa, which was consistent with the predicted MW of rTsCTL (the cDNA clone was 24 kDa and GST-tag was 26 kDa) (Figure 1A). Western blotting analysis revealed that rTsCTL was recognized by anti-rTsCTL serum, anti-GST-tag serum and infection serum (Figure 1B), but not by normal serum.

### Cell viability assayed with the CCK-8 test

**Figure 2:**
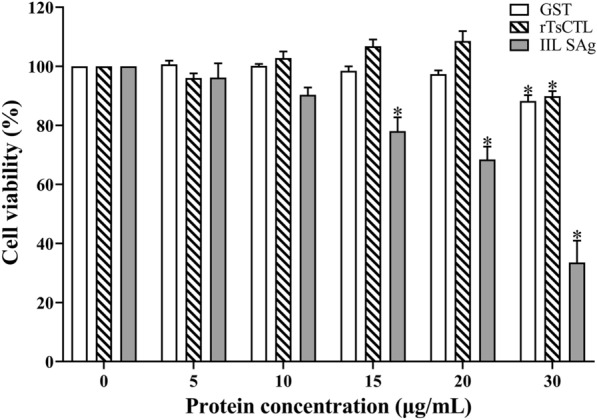
**The effect of rTsCTL, IIL SAg and GST on the viability of Caco-2 cells.** Caco-2 cells were incubated with different concentrations of GST, rTsCTL and IIL SAg for 24 h, and cell viability was detected. Cell viability = (OD values of test group − OD values of blank control)/(OD values of GST tag control − OD values of blank control) × 100%. *P < 0.001 indicates an obvious reduction of cell activity compared to the blank control group.

The results of the CCK-8 test show that when Caco-2 cells were incubated with various doses of proteins for 24 h, a significant decrease of cell viability was observed in the 15–30 µg/mL IIL SAg (*F* = 86.92, *P*_15_ < 0.001, *P*_20_ < 0.0001, *P*_30_ < 0.0001), and 30 µg/mL rTsCTL and GST (*F*_rTsCTL_ = 32.17, *P* < 0.001; *F*_GST_ = 39.02, *P* < 0.0001), respectively, but not in the other dose groups (Figure 2). Consequently, 5 µg/mL rTsCTL, IIL SAg and GST were used in the subsequent experiment.

### Co-localization of rTsCTL with syndecan-1 in Caco-2 cells

**Figure 3:**
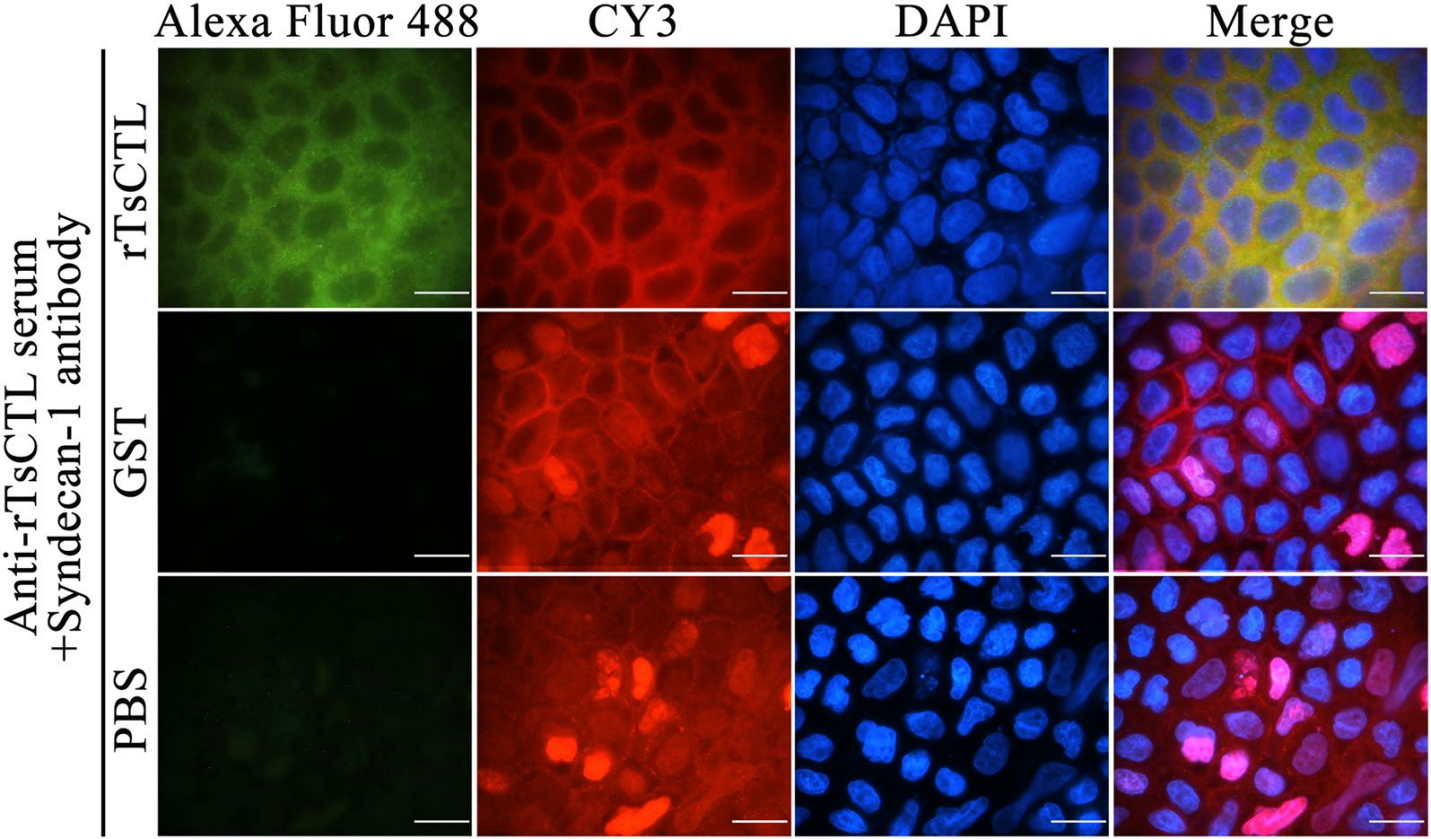
**Immunofluorescence co-localization of rTsCTL and syndecan-1.** Alexa Fluor 488: Caco-2 cells were incubated with rTsCTL, GST and PBS, then probed by anti-rTsCTL serum, and stained by Alexa Fluor 488-conjugated anti-mouse IgG. CY3: Caco-2 cells were probed by anti-syndecan-1 antibody, and stained by CY3-conjugated anti-rabbit IgG. DAPI: cell nuclei were stained by DAPI as blue; scale bars: 40 μm.

The IFA results show that green fluorescence on Caco-2 cells incubated with rTsCTL was observed using anti-rTsCTL serum. Red fluorescence on Caco-2 cells incubated with rTsCTL was also found by anti-syndecan-1 antibody. After integration, both rTsCTL and syndecan-1 were co-localized on the cell membrane as orange (Figure 3).

### Specific binding of rTsCTL to syndecan-1 in Caco-2 cells

**Figure 4:**
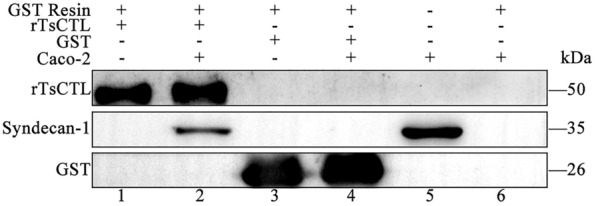
**Binding of rTsCTL to syndecan-1 in Caco-2 cells assessed by GST pull-down and Western blot analysis.** Lane 1: GST resins + rTsCTL; Lane 2: GST resins + rTsCTL + Caco-2 cell proteins; Lane 3: GST resins + GST; Lane 4: GST resins + GST + Caco-2 cell proteins; Lane 5: The lysates of Caco-2 cells; Lane 6: GST resins + Caco-2 cell proteins.

The results of GST pull-down and Western blot show that rTsCTL was bound to syndecan-1 in Caco-2 cells; whereas GST-tag protein and blank resins failed. The results validated that rTsCTL could specifically bind to syndecan-1 deployed on the Caco-2 cells in vitro (Figure 4).

**Figure 5:**
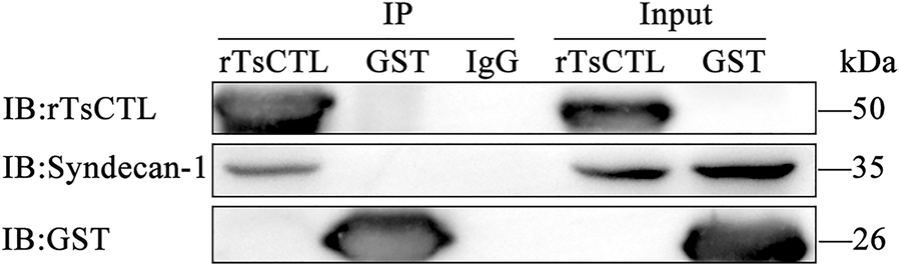
**Binding of rTsCTL and syndecan-1 in Caco-2 cells detected by Co-IP.** rTsCTL: IP samples of Caco-2 cellular protein incubated with anti-rTsCTL serum; GST: IP samples of Caco-2 cell proteins incubated with anti-GST serum; IgG: IP samples of Caco-2 cell proteins incubated with normal mouse IgG; Input: input rTsCTL, Caco-2 cell lysate and GST were examined by using anti-rTsCTL serum, anti-syndecan-1 antibody and anti-GST serum; IP: immunoprecipitation; IB: immunoblotting.

The results from the Co-IP assay show that agarose conjugated with anti-rTsCTL serum was able to precipitate the complexes of GST-rTsCTL and syndecan-1. However, the GST control could not precipitate syndecan-1. Moreover, normal murine IgG did not precipitate rTsCTL and syndecan-1 (Figure 5). The results indicate that there was a specific binding and interaction between rTsCTL and natural syndecan-1 in Caco-2 cells.

### Binding of rTsCTL to syndecan-1 reduced expression of gut TJ proteins via activation of the STAT3 pathway

**Figure 6:**
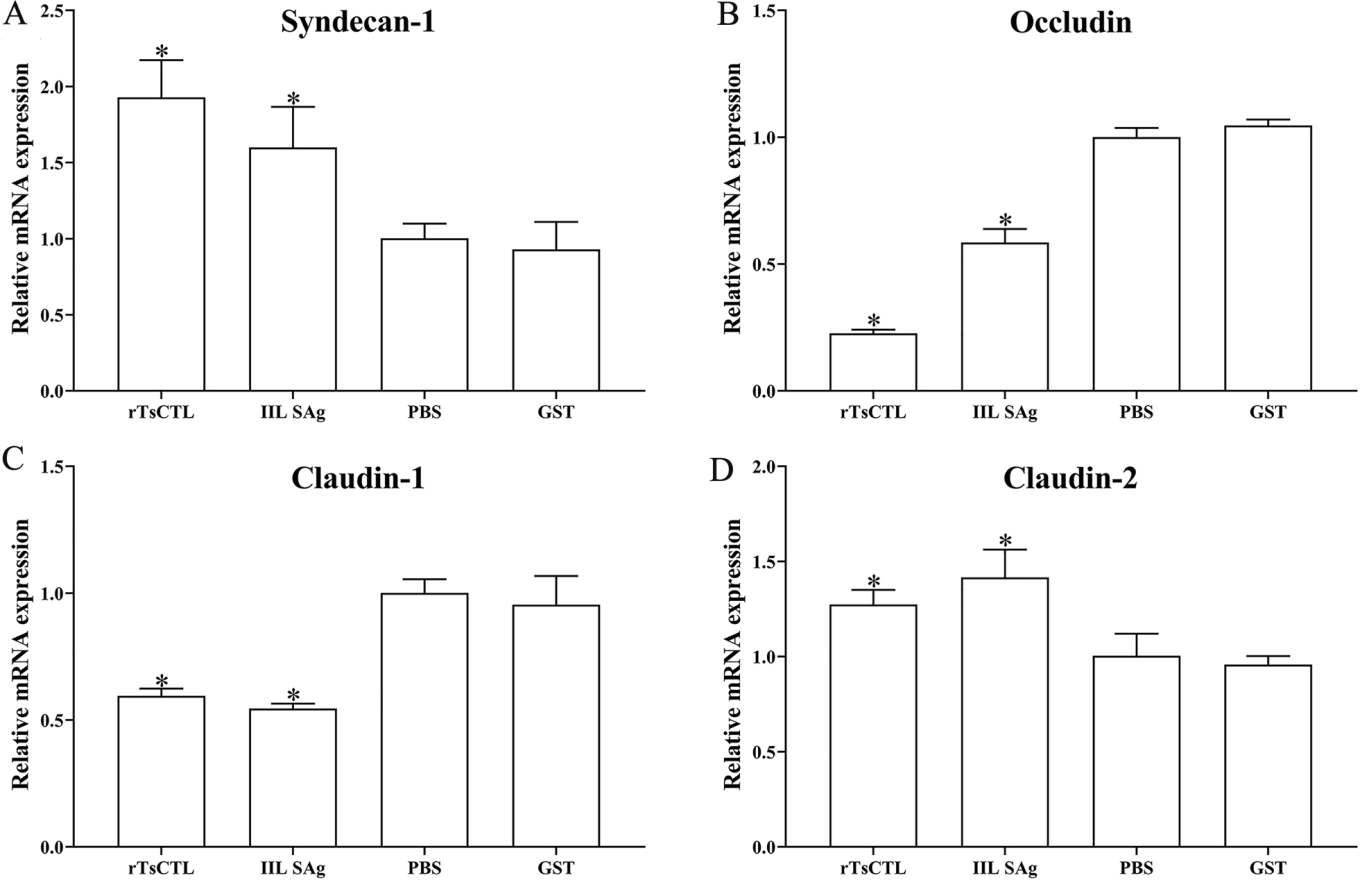
**qPCR analysis of the transcription levels of syndecan-1 and TJs in Caco-2 cells incubated with rTsCTL.** Caco-2 cells were incubated with rTsCTL (5 µg/mL), and IIL SAg and GST tag protein were respectively used as a positive or negative control. The transcription levels of syndecan-1 (**A**), occludin (**B**), claudin-1 (**C**), and claudin-2 (**D**) were analyzed by qPCR. The transcription levels were calculated with the 2^−ΔΔCt^ method. β-Actin was used as an internal control. *P < 0.05 compared to the PBS group.

**Figure 7:**
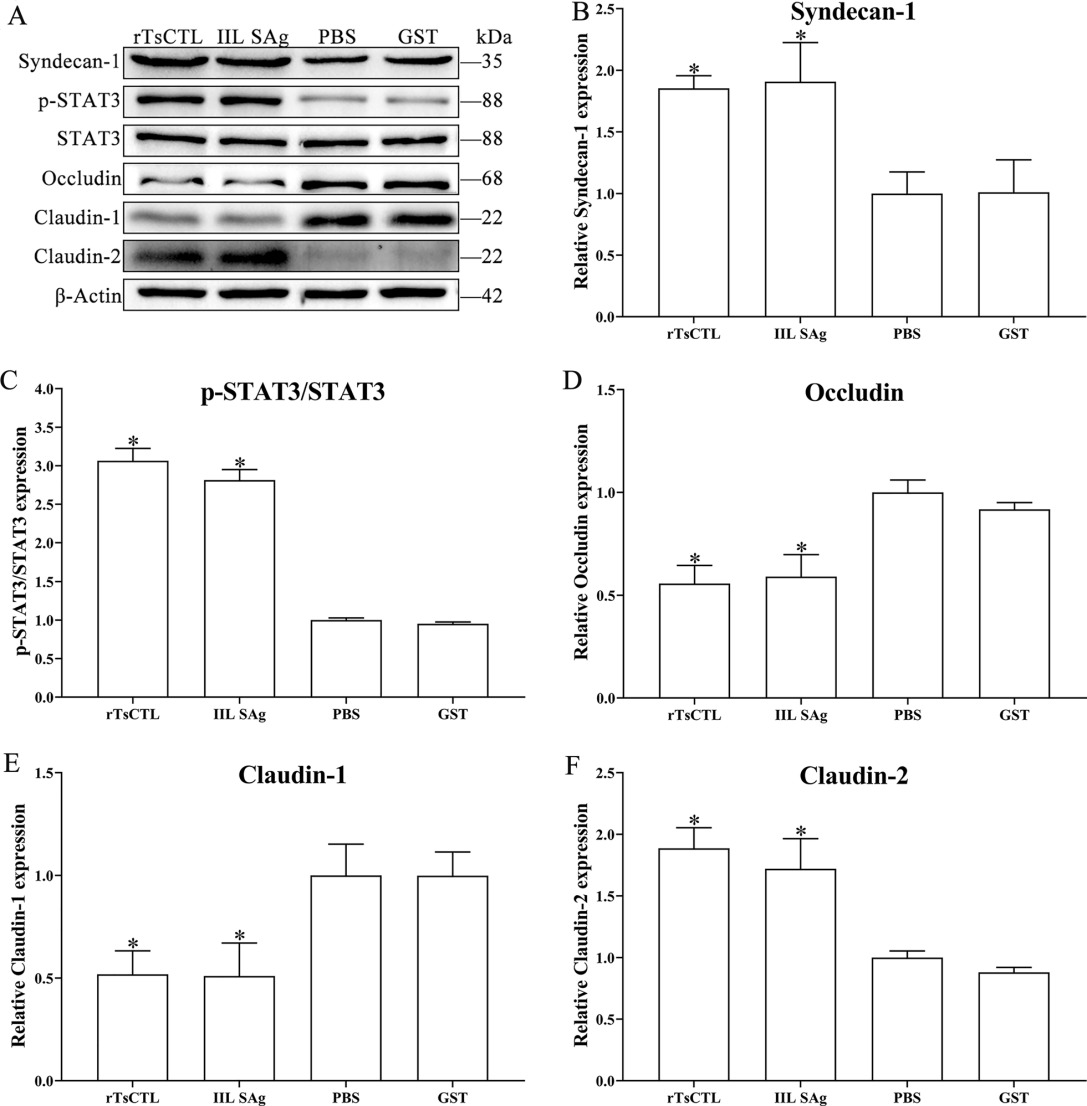
**Western blotting of expression levels of syndecan-1, p-STAT3 and TJs in Caco-2 cells incubated with rTsCTL. A** Caco-2 cells were incubated with rTsCTL (5 µg/mL), and IIL SAg and GST tag protein were respectively used as a positive or negative control. The expression levels of syndecan-1, p-STAT3, STAT3, occludin, claudin-1, and claudin-2 were analyzed by Western blotting, and β-Actin was used as an internal reference control. **B**–**F** Densitometric analysis of protein bands obtained in panel (**A**) for syndecan-1 (**B**), p-STAT3/STAT3 (**C**), occludin (**D**), claudin-1 (**E**) and claudin-2 (**F**) relative to the β-Actin band *P < 0.01 compared to the PBS group.

After Caco-2 cells were stimulated by rTsCTL, compared to the PBS group, the transcription levels of syndecan-1 and claudin-2 evidently increased (syndecan-1: *F* = 16.05, *P* < 0.01; claudin-2: *F* = 13.33, *P* < 0.05), but the transcription levels of occludin and claudin-1 obviously decreased (occludin: *F* = 364.6, *P* < 0.0001; claudin-1: *F* = 33.26, *P* < 0.001) (Figure 6). Meanwhile, the expression levels of syndecan-1, p-STAT3 and claudin-2 significantly increased (syndecan-1: *F* = 14.49, *P* < 0.01; p-STAT3: *F* = 340.3, *P* < 0.0001; claudin-2: *F* = 33.26, *P* < 0.001), but the expression levels of occludin and claudin-1 decreased (occludin: *F* = 25.86, *P* < 0.001; claudin-1: *F* = 12.49, *P* < 0.01) (Figure 7). The results suggest that binding of rTsCTL to syndecan-1 in Caco-2 cells reduced expression of occludin and claudin-1, increased claudin-2 expression, and impaired the integrity of the gut epithelial barrier.

### Inhibitors suppressed rTsCTL binding to syndecan-1, blocked STAT3 pathway activation and reduced expression of TJ proteins

**Figure 8:**
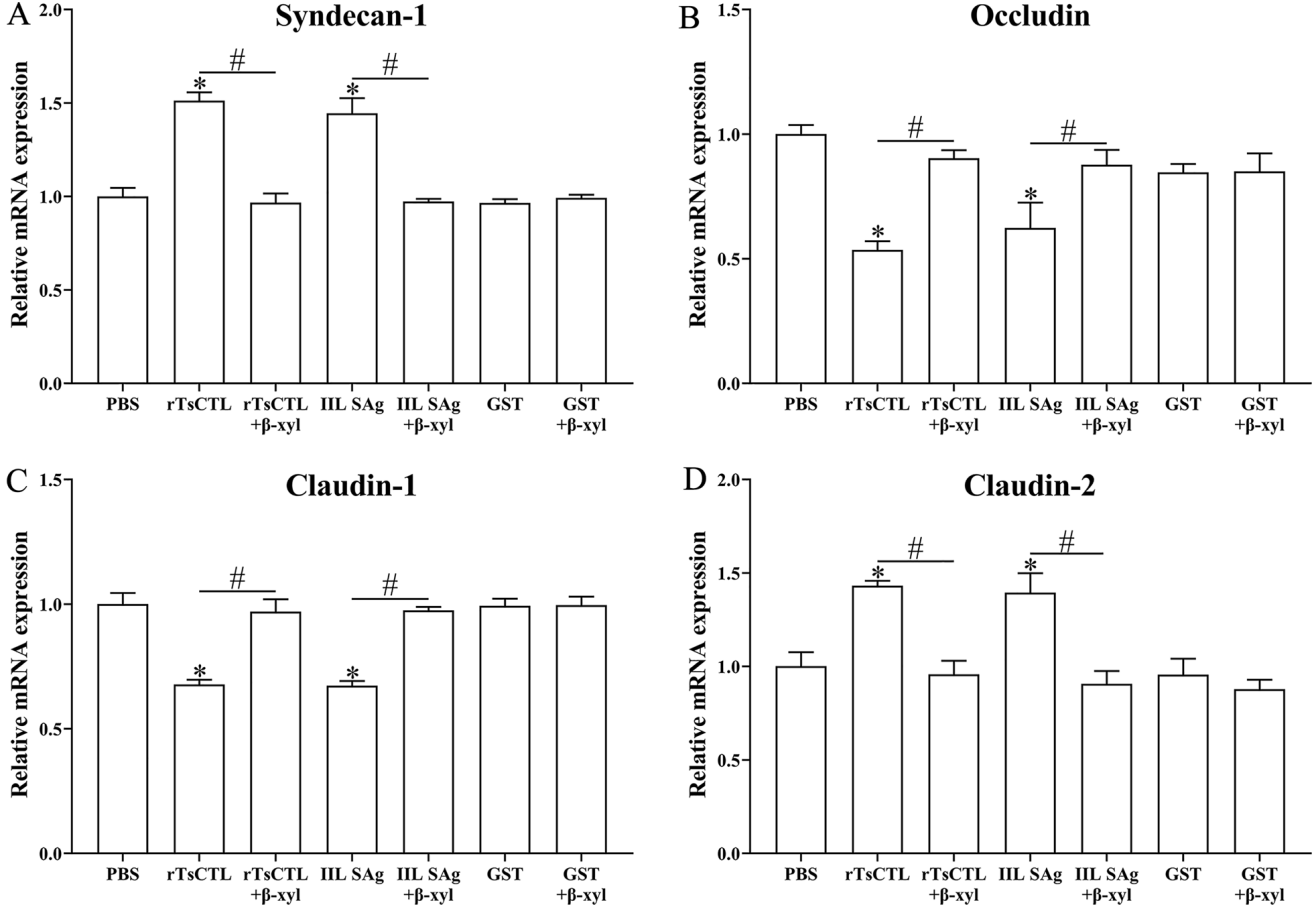
**qPCR analysis of transcription levels of syndecan-1 and TJs in Caco-2 cells after β-xyloside treatment.** Caco-2 cells were pretreated with β-xyloside (5 mM) and then incubated with rTsCTL (5 µg/mL), and IIL SAg and GST tag protein were respectively used as a positive or negative control. The transcription levels of syndecan-1 (**A**), occludin (**B**), claudin-1 (**C**), and claudin-2 (**D**) were ascertained by qPCR. The transcription levels were calculated with the 2^−ΔΔCt^ method. β-Actin was used as an internal control. *P < 0.01 compared to the PBS group. ^#^*P* < 0.001 compared between two groups.

**Figure 9:**
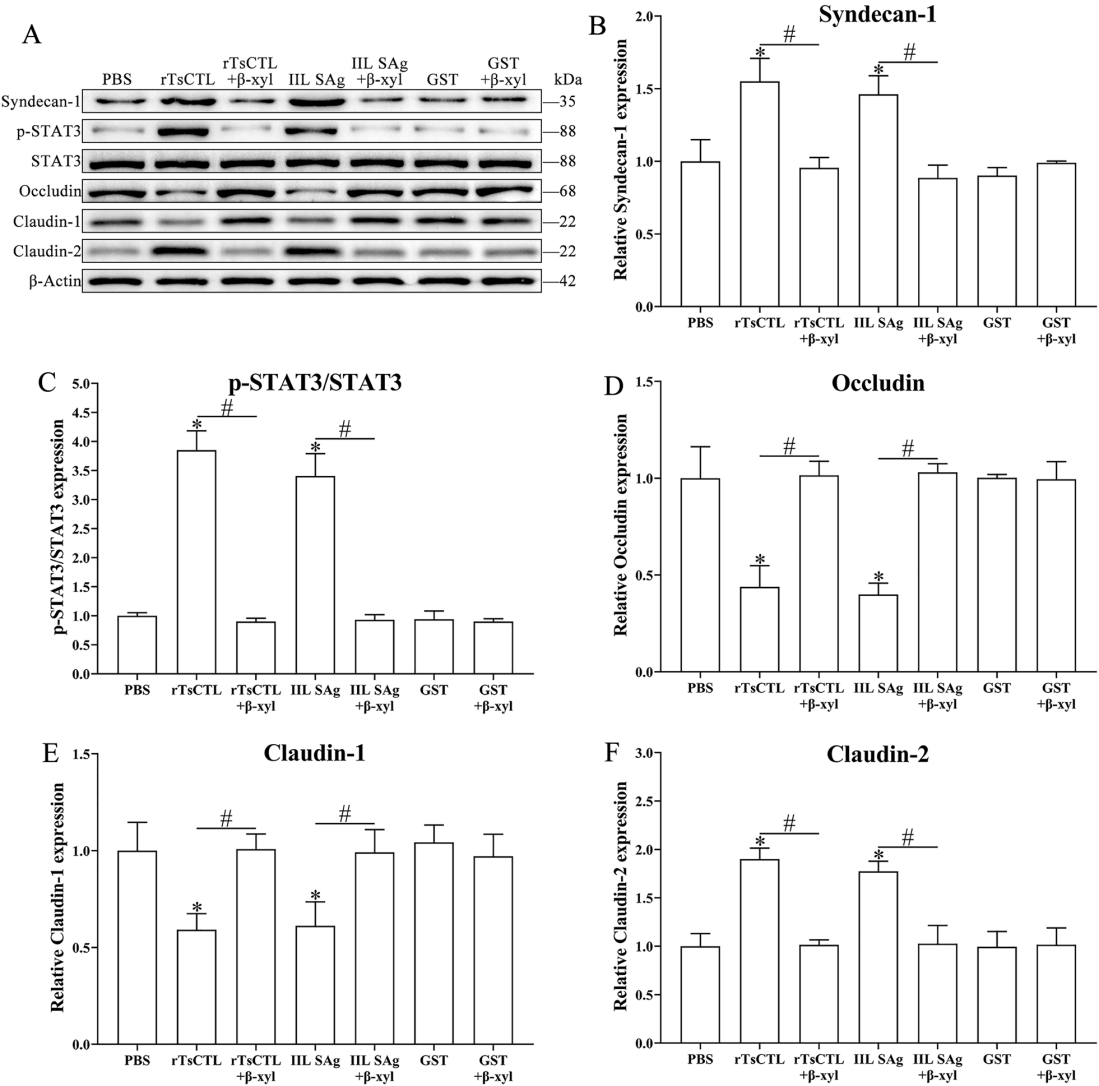
**Western blot analysis of expression levels of syndecan-1, p-STAT3 and TJs in Caco-2 cells after β-xyloside treatment. A** Caco-2 cells were pretreated with β-xyloside (5 mM) and then incubated with rTsCTL (5 µg/mL), and IIL SAg and GST tag protein were respectively used as a positive or negative control. The expression levels of syndecan-1, p-STAT3, STAT3, occludin, claudin-1, and claudin-2 were analyzed by Western blot, and β-Actin was used as an internal reference control. **B**–**F** Densitometric analysis of the bands obtained in (**A**) panel for syndecan-1 (**B**), p-STAT3/STAT3 (**C**), occludin (**D**), claudin-1 (**E**) and claudin-2 (**F**) relative to the β-Actin band *P < 0.01 compared with PBS. ^#^*P* < 0.01 compared between two groups.

After Caco-2 cells were pre-treated with β-xyloside and Stattic, qPCR and Western blotting results show that β-xyloside and Stattic abrogated rTsCTL’ disruption on TJ in the Caco-2 cell monolayer. Compared to the Caco-2 cells incubated with only rTsCTL, β-xyloside obviously inhibited the transcription levels of syndecan-1 and claudin-2 in Caco-2 cells incubated with rTsCTL (syndecan-1: *t* = 14.28, *P* < 0.001; claudin-2: *t* = 10.61, *P* < 0.001), but distinctly up-regulated the transcription levels of occludin and claudin-1 (occludin: *t* = 13.34, *P* < 0.001; claudin-1: *t* = 9.52, *P* < 0.001) (Figure 8). Moreover, β-xyloside also significantly decreased the expression levels of syndecan-1, p-STAT3, and claudin-2 (syndecan-1: *t* = 5.968, *P* < 0.001; p-STAT3: *t* = 15.1, *P* < 0.001; claudin-2: *t* = 12.31, *P* < 0.001), and increased the expression of occludin and claudin-1 (occludin: *t* = 7.604, *P* < 0.05; claudin-1: *t* = 6.328, *P* < 0.01) (Figure 9). The results suggest that β-xyloside obviously inhibited rTsCTL binding to syndecan-1 in Caco-2 cells, and abrogated the regulation of rTsCTL binding to syndecan-1 on expression of gut tight junctions.

**Figure 10:**
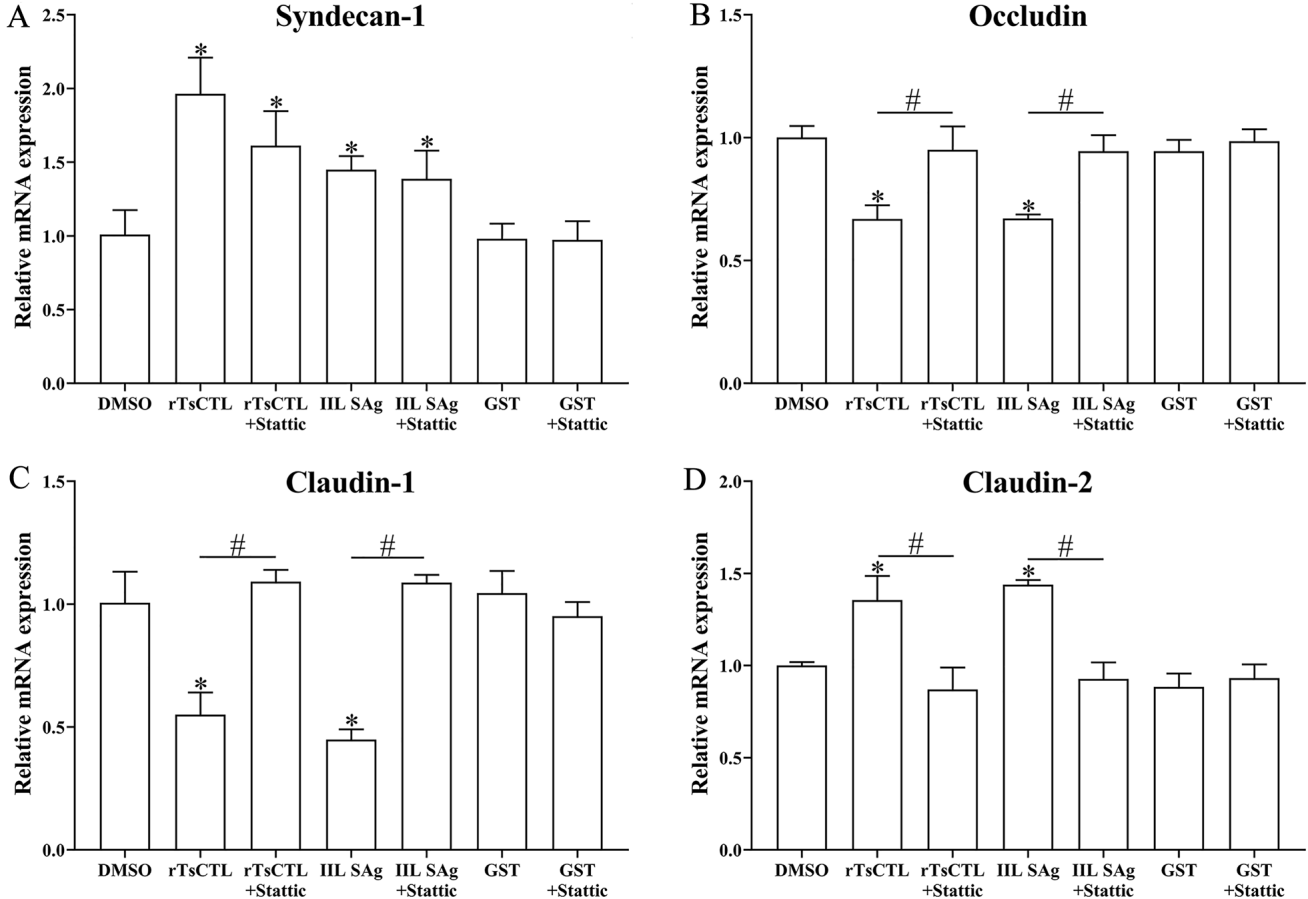
**qPCR analysis of transcription levels of syndecan-1 and TJs in Caco-2 cells treated with Stattic.** Caco-2 cells were pre-treated with Stattic (10 µM) and then incubated with rTsCTL (5 µg/mL), 0.1% DMSO (Stattic solvent) was used as a negative control. The transcription levels of syndecan-1 (**A**), occludin (**B**), claudin-1 (**C**), and claudin-2 (**D**) were assessed by qPCR. The transcription levels were calculated with the 2^−ΔΔCt^ method. β-Actin was used as an internal control. *P < 0.05 compared to the DMSO group. ^#^*P* < 0.05 compared between two groups.

**Figure 11:**
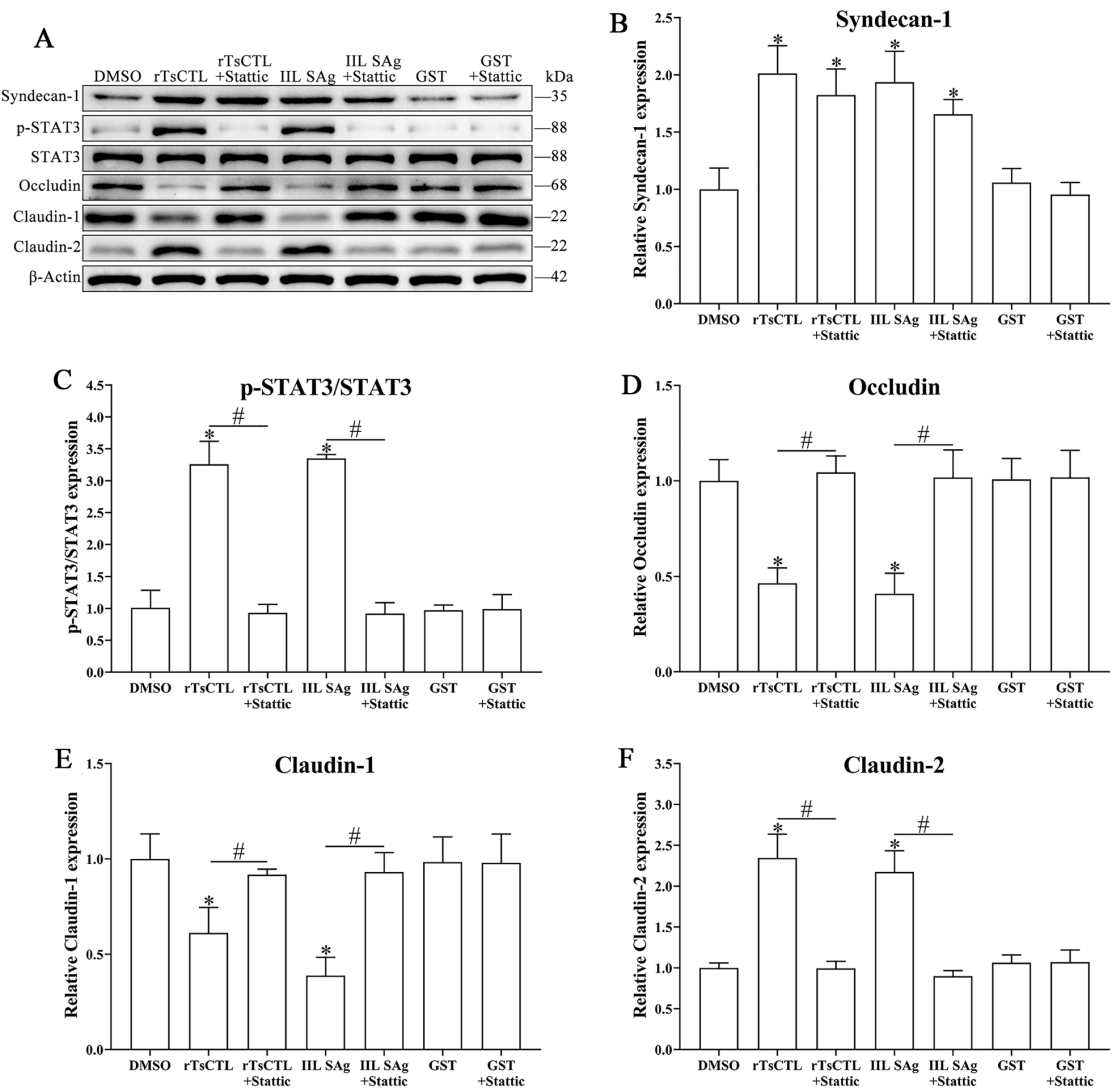
**Western blotting analysis of expression levels of syndecan-1, p-STAT3 and TJ in Caco-2 cells after Stattic treatment. A** Caco-2 cells were pre-treated with Stattic (10 µM) and then incubated with rTsCTL (5 µg/mL), and 0.1% DMSO (Stattic solvent) was used as a negative control. The expression levels of syndecan-1, p-STAT3, STAT3, occludin, claudin-1, and claudin-2 were ascertained by Western blotting, and β-Actin was used as an internal reference control. **B**–**F** Densitometric analysis of the bands obtained in (**A**) for syndecan-1 (**B**), p-STAT3/STAT3 (**C**), occludin (**D**), claudin-1 (**E**) and claudin-2 (**F**) relative to the β-Actin band. *P < 0.01 relative to the DMSO group. ^#^*P* < 0.01 compared between two groups.

To confirm further the effect of STAT3 pathway activation on TJ expression, Caco-2 cells were also pretreated using a STAT3 inhibitor, Stattic. The results reveal that Stattic partially inhibited rTsCTL-activated transcription level of claudin-2 in Caco-2 cells (*t* = 4.738, *P* < 0.01), Stattic abrogated and restored the rTsCTL-suppressed transcription level of occludin and claudin-1 (occludin: *t* = 4.423, *P* < 0.05; claudin-1: *t* = 9.165, *P* < 0.001) (Figure 10). Western blot results show that Stattic significantly inhibited rTsCTL-stimulated expression level of claudin-2 and p-STAT3 in Caco-2 cells (claudin-2: *t* = 7.764, *P* < 0.01; p-STAT3: *t* = 10.47, *P* < 0.001); however, Stattic abrogated and regained prominently the rTsCTL-suppressed expression level of occludin and claudin-1 (occludin: *t* = 8.536, *P* < 0.01; claudin-1: *t* = 3.872, *P* < 0.01) (Figure 11). The results further confirmed that the binding of rTsCTL to syndecan-1 activated the STAT3 pathway and reduced the expression of the TJ proteins (occludin and claudin-1), consequently destroying the intestinal epithelial barrier function.

### Binding of rTsCTL to syndecan-1 mediated larval invasion of Caco-2 cells

**Figure 12:**
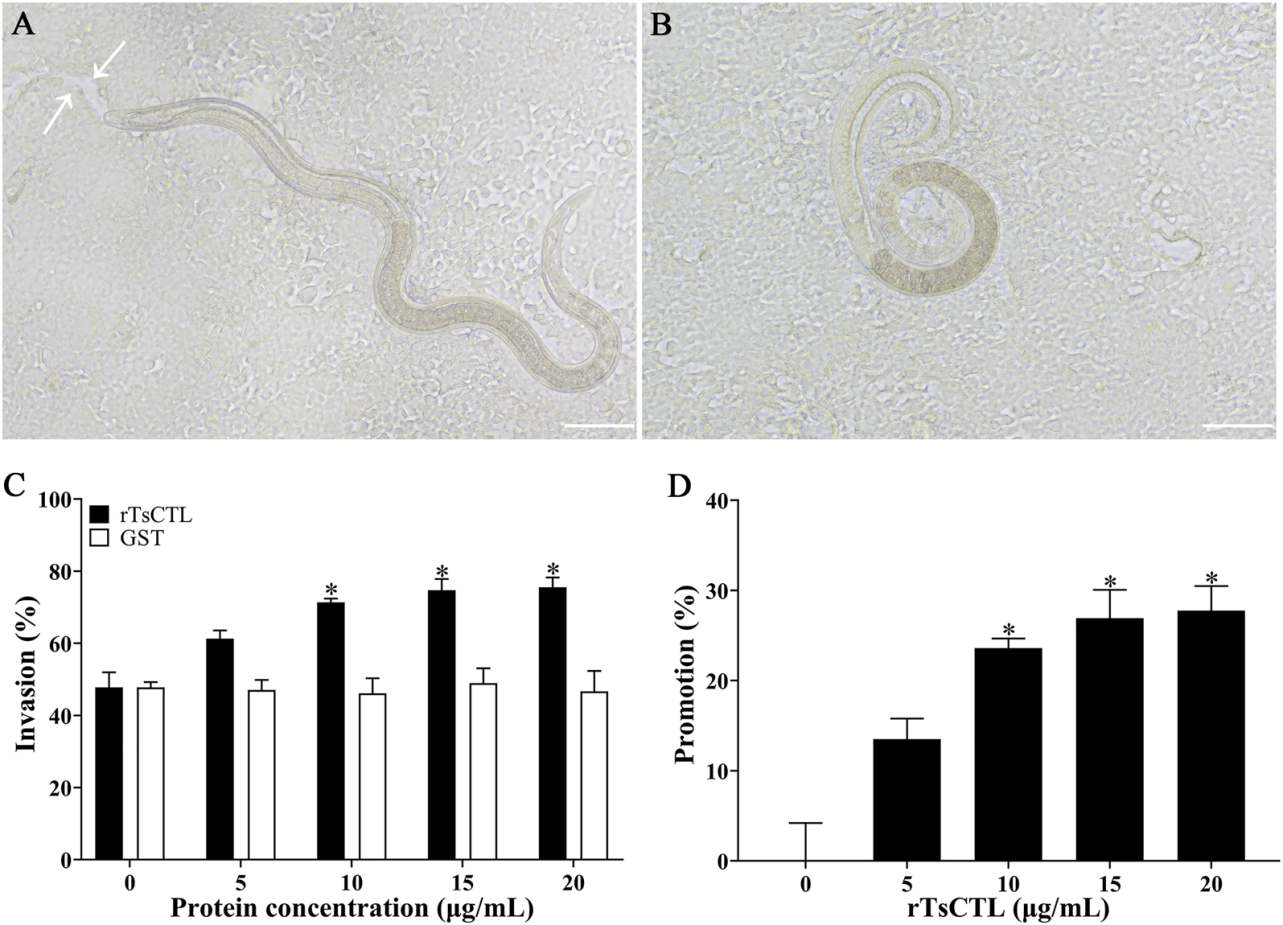
**Facilitation of rTsCTL on larval invasion of Caco-2 cells. A** The invaded larva was mobile and migratory in the monolayer (the white arrows showed the migratory trace). **B** Non-invaded larva was coiled on the Caco-2 surface. **C** and **D** rTsCTL accelerated IIL invasion into Caco-2 cells. Scale bars: 100 μm. *P < 0.05 compared to the GST or PBS control group. Promotion (%) = Invasion rate of the experimental group − average invasion rate of the PBS control group.

The in vitro larval invasion assay shows that rTsCTL significantly promoted the larval invasion of Caco-2 cells in an rTsCTL dose-dependent manner (*r* = 0.858, *P* < 0.0001), and the rTsCTL promotion on larval invasion was enhanced with the increase of rTsCTL dose (*F* = 49.76, *P* < 0.0001) (Figure 12).

### Inhibitors abrogated the rTsCTL promotion role on larval invasion of Caco-2 cells

**Figure 13:**
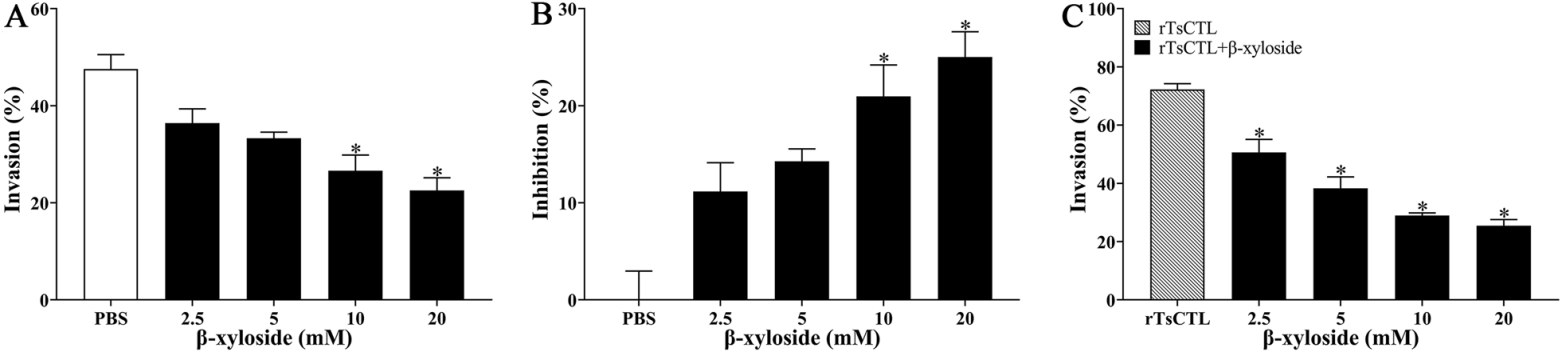
**β-xyloside inhibited larval invasion of Caco-2 cells and abrogated rTsCTL facilitative role on the invasion. A** and **B** 10 and 20 mM β-xyloside significantly inhibited larval invasion of Caco-2 cells. **C** 2.5–20 mM β-xyloside significantly inhibited and abrogated the rTsCTL promotion role on larval invasion of Caco-2 cells. * *P* < 0.05 compared to the PBS group or only rTsCTL group. Inhibition (%) = average invasion rate of the PBS control group − invasion rate of the experimental group.

The suppressive role of β-xyloside on larval invasion of Caco-2 cells was observed; the suppression of 10 and 20 mM β-xyloside on the invasion was 20.97 and 25.01%, compared to the PBS group (*χ*^*2*^_10_ = 6.124, *χ*^*2*^_20_ = 7.128, *P* < 0.05). The suppressive role had a correlation with the dose of β-xyloside (*r* = 0.890, *P* < 0.001) and exhibited an elevating trend with the increase of β-xyloside dose (*F* = 38.469, *P* < 0.001) (Figure 13A, B). Additionally, the rTsCTL promotion on larval invasion was significantly suppressed and abrogated by 2.5–20 mM β-xyloside, as demonstrated by a reduction of 21.65, 33.96, 43.25 and 46.79% (χ^2^_2.5_ = 5.538, *P* < 0.05; χ^2^_5_ = 14.345, χ^2^_10_ = 21.913, χ^2^_20_ = 25.849, *P* < 0.0001), the suppressive role of β-xyloside also had a correlation with the dose of β-xyloside, (*r* = 0.950, *P* < 0.001) and exhibited an elevating trend with the increase of β-xyloside dose (*F* = 119.841, *P* < 0.001) (Figure 13C).

**Figure 14:**
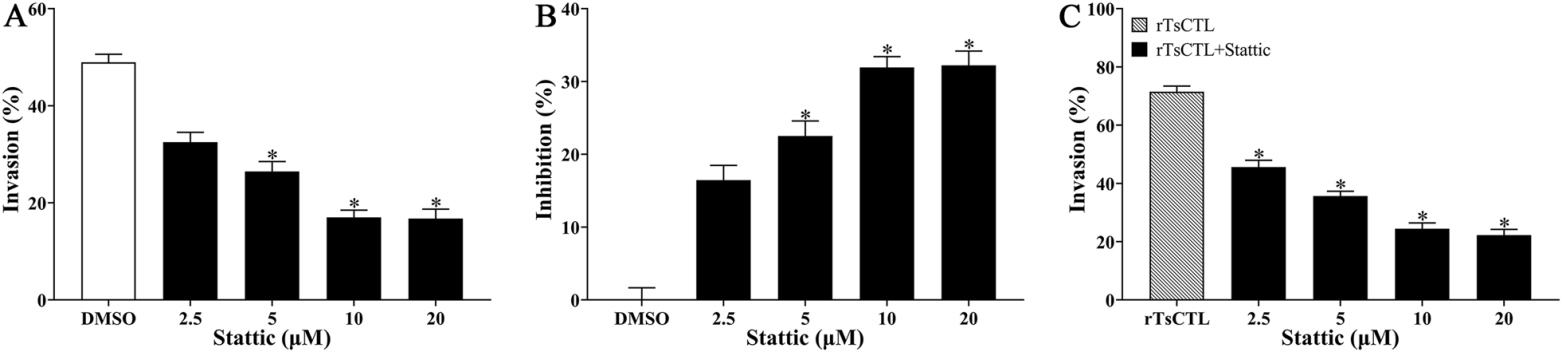
**Stattic inhibited larval invasion of Caco-2 cells and abrogated rTsCTL facilitative role on the invasion. A** and **B** Stattic at 5, 10 and 20 µM significantly inhibited larval invasion of Caco-2 cells. **C** 2.5–20 µM Stattic significantly inhibited and abrogated the rTsCTL promotion role on the in vitro larval invasion of Caco-2 cells. *P < 0.05 compared to the DMSO or only rTsCTL group. Inhibition (%) = Average invasion rate of the DMSO control group − invasion rate of the experimental group.

Meanwhile, Stattic also had a suppressive role on larval invasion of Caco-2 cells, the inhibition rate of 5, 10 and 20 µM Stattic on larval invasion was 22.51, 31.94, and 32.21%, respectively, compared to the DMSO group (χ^2^_5_ = 5.543, *P* < 0.05; χ^2^_10_ = 11.484, *P* < 0.05; χ^2^_20_ = 12.749, *P* < 0.0001). The suppression had a correlation with the dose of Stattic (*r* = 0.823, *P* < 0.01) and exhibited an elevating trend with the increase of Stattic dose (*F* = 154.029, *P* < 0.001) (Figure 14A, B). Moreover, Stattic also evidently inhibited and abrogated the rTsCTL facilitative effect on larval invasion of Caco-2 cells, the inhibition rate of 2.5–20 µM Stattic was 25.88, 35.84, 47.04 and 49.26% respectively, compared to the rTsCTL group alone (χ^2^_2.5_ = 7.486, *P*_5_ < 0.01; χ^2^ = 15.088, χ^2^_10_ = 27.074, χ^2^_20_ = 27.827, *P* < 0.0001). The inhibition and abrogation had a correlation with the dose of Stattic (*r* = 0.942, *P* < 0.001) and exhibited an elevating trend with the increase of Stattic dose (*F* = 302.481, *P* < 0.001) (Figure 14C).

## Discussion

C-type lectin (CTL) is a family of proteins containing one or more carbohydrate recognition domains (CRD) and binding to a variety of ligands in the presence of Ca^2+^. The CTL can be used as pattern recognition receptors (PRR) to participate in a variety of immune processes, and plays an important role in parasite adhesion, invasion and immune evasion [53]. A C-type lectin was found in the secretory products of *Toxocara canis* infectious larvae. It selectively binds to ligands on the surface of dog MDCK cells in a calcium-dependent manner in vitro [54]. The C-type lectin (CD209a) on host dendritic cells recognizes glycoproteins on the surface of *Schistosoma* eggs and mediates the invasion of *Schistosoma* into host connective tissues [55]. The C-type lectin CpCTL of *Cryptosporidium parvum* mediates the parasite invasion and infection of IEC [17]. However, there are a few reports on the function and mechanism of *T. spiralis*-derived C-type lectin in the literature.

A novel TsCTL is a surface and secretory protein which is expressed at various *T. spiralis* developmental stages and highly expressed at the invasive stage IIL; TsCTL directly contacted and interacted with the host intestinal epithelium [18, 27]. TsCTL as a surface and secretory antigen was also early exposed to the host’s immune system and could trigger the generation of specific anti-*Trichinella* IgG antibodies, the serum IgG antibodies in mice infected with 100 ML were detectable by ELISA and Western blotting as early as 10 dpi [56, 57]. Therefore, the purified rTsCTL protein was recognized by *T. spiralis*-infected mouse serum as shown in Figure 1B. Additionally, the purified rTsCTL might contain certain bacterial components; whereas mouse infection sera possibly had anti-bacterium antibodies, consequently other several weak bands beside the rTsCTL with 50 kDa were also indistinctly probed by murine infection sera [31].

Previous studies have shown that rTsCTL promoted the IIL larvae invasion into host’ IEC, but the mechanism of rTsCTL promotion on the IIL invasion was unclear [20]. The C-type lectin of *Cryptosporidium parvum* mediated the *Cryptosporidium* attachment and infection to IEC by interacting with heparan sulfate proteoglycans (HSPG) on the IEC [17]. Syndecan-1 is a type I integral membrane proteoglycan, which belongs to the HSPG family and is mainly expressed on the epithelial surface and extracellular matrix. It acts as a primary role for maintaining cell morphology, establishing intercellular adhesion and regulating the intestinal mucosal epithelial barrier [23]. But, the interaction between TsCTL and syndecan-1 in IEC has not been reported up to now.

To investigate whether TsCTL binds to SDC-1 on IEC, we selected pGEX-4T-1 as the expression vector of rTsCTL. The whole sequence of TsCTL cloned and expressed in this study had the tac promoter and GST tag sequence. The rTsCTL was expressed in large amounts in *E. coli* by using the GST fusion expression system and increased the solubility of rTsCTL protein. The GST-rTsCTL protein was purified by GST purification resins, and the purified GST-rTsCTL was recognized by infected serum, anti-rTsCTL serum and anti-GST serum, indicating that GST-rTsCTL had good antigenicity. IFA results show that rTsCTL and syndecan-1 were co-localized on the membrane of Caco-2 cells. GST pull-down is an intuitive, fast, and simple screening technique for the identification of protein–protein or protein–ligand interactions by immobilizing GST fusion proteins on GST-purified resins [58]. In this study, GST-rTsCTL was immobilized on the GST-purified resins to precipitate the interacted proteins in Caco-2 cells. Western blot shows that GST-rTsCTL could bind to the ligand syndecan-1 in Caco-2 cells. However, the GST-tag protein and blank GST-purified resins did not bind to syndecan-1, indicating that rTsCTL could specifically bind to syndecan-1 in vitro. Co-IP was also used to verify the binding of rTsCTL to syndecan-1 in Caco-2 cells in the natural state. Co-IP is a technology that uses antibodies to capture target proteins, and their interacting proteins or complexes from samples, which can specifically enrich the target proteins tested. Non-denaturing conditions were used to preserve the intracellular state of the interacting proteins [59, 60]. Our Co-IP results show that anti-rTsCTL serum-conjugated beads could precipitate rTsCTL carrying GST tag and syndecan-1 complex, while the GST alone control did not precipitate syndecan-1, and normal murine IgG did not precipitate rTsCTL and syndecan-1. Therefore, our results demonstrate that rTsCTL specifically bound and interacted with syndecan-1 in Caco-2 cells.

Syndecan-1, one of heparan sulfate proteoglycan, is essential for maintaining normal cell morphology, interacting with extracellular and intracellular protein libraries, and mediating signal transduction in response to environmental stimuli [61]. Syndecan-1 significantly regulated the expression of ZO-1 and occludin by activating STAT3. Additionally, ZO-1 and occludin were found to bind to each other, and their repression may induce syndecan-1 up-regulation [62]. The results of qPCR and Western blot show that the expression of syndecan-1 was up-regulated in Caco-2 cells after rTsCTL stimulation, which mediated STAT3 phosphorylation, causing a decrease in expression levels of TJ proteins (occludin and claudin-1), and increasing the expression level of claudin-2. Tight junctions, since the most important intercellular junctions, determine intestinal epithelial permeability and maintain the physiological function of the intestinal barrier [63]. Occludin and claudins play a crucial role in maintaining cell polarity and intestinal epithelial barrier [64]. Occludin acts as a closed paracellular space, and overexpression of occludin enhances epithelial barrier function in vitro [65, 66]. Claudin-1 plays a blocking role in the intestinal mucosal barrier and reduces the permeability of cellular bypass. Claudin-2, known as a pore-forming protein, plays an important role in cell bypass pore formation, the cation permeability, ion size selectivity and water transport. Previous studies show that claudin-2 overexpression or up-regulation of claudin-2 expression increased intestinal permeability, and deteriorated colitis [67]. Therefore, rTsCTL binding and interaction with syndecan-1 on Caco-2 cells activated STAT3 phosphorylation, reduced TJ protein expression, impaired the integrity of the gut epithelium barrier, and finally mediated *T. spiralis* larval invasion of the gut mucosa.

In the life cycle of *T. spiralis*, the IIL invasion into intestinal mucosa is the key step for successfully infecting the host [68]. Previous studies revealed that the IIL could penetrate into Caco-2, HCT-8, T84 and other epithelial cells [51, 69]. In order to ascertain the role of rTsCTL binding to syndecan-1 in the IIL invasion of the intestinal epithelia, an in vitro invasion model of Caco-2 cells was used in this study. The in vitro larval invasion results show that rTsCTL significantly promoted the IIL invasion of Caco-2 monolayer, and the rTsCTL promoting invasion was enhanced with the increase of rTsCTL dose. Moreover, β-xyloside and Stattic inhibited the IIL invasion of Caco-2 cells, and significantly abrogated the rTsCTL promotion on larval invasion of Caco-2 cells. The results further verified that rTsCTL binding to syndecan-1 on gut epithelium activated STAT3 phosphorylation, reduced TJ expression, disrupted the integrity of the gut epithelium barrier, and consequently mediated the IIL invasion of gut mucosa [6, 39].

In conclusion, our results show that there was a specific binding and interaction between rTsCTL and syndecan-1 in Caco-2 cells. rTsCTL binding to syndecan-1 increased the expression of syndecan-1 and claudin-2, and reduced the expression of occludin and claudin-1 in Caco-2 cells via the STAT3 pathway. β-Xyloside (a syndecan-1 synthesis inhibitor) and Stattic (a STAT3 inhibitor) significantly inhibited rTsCTL binding to syndecan-1 in Caco-2 cells and activation of the STAT3 pathway, abrogated the effects of rTsCTL on expression of gut tight junctions, and impeded larval invasion. The results demonstrate that rTsCTL binding to syndecan-1 in Caco-2 cells activated the STAT3 pathway, reduced expression of tight junction proteins, impaired the integrity of intestinal epithelium barrier, and mediated the *T. spiralis* larval penetration of intestinal mucosa. TsCTL might be a candidate molecule target of preventive vaccines against *T. spiralis* invasion and infection.

## Abbreviations

AW: adult worm
Co-IP: co-immunoprecipitation
CRD: carbohydrate recognition domain
CTL: C-type lectin
DAB: 3,3′-Diaminobenzidine tetrahydrochloride
DAPI: 4′,6-Diamidino-2- phenylindole
DMEM: Dulbecco’s modified eagle medium
hpi: hours post-infection
HRP: horseradish peroxidase
HSPG: heparan sulfate proteoglycans
IECs: intestinal epithelium cells
IFA: immunofluorescence assay
IIL: intestine infectious larvae
ML: muscle larvae
MW: molecular weight
NBL: newborn larvae
NC: nitrocellulose
PRR: pattern recognition receptors
PVDF: polyvinylidene difluoride
qPCR: real-time quantitative PCR
SAg: soluble antigens
SDC-1: syndecan-1
TBST: Tris-buffered saline containing 0.05% Tween
TJs: tight junctions
TsCTL: *Trichinella spiralis* C-type lectin
